# Long-term ambient hydrocarbons exposure and incidence of ischemic stroke

**DOI:** 10.1371/journal.pone.0225363

**Published:** 2019-12-04

**Authors:** Han-Wei Zhang, Victor C. Kok, Shu-Chun Chuang, Chun-Hung Tseng, Chin-Teng Lin, Tsai-Chung Li, Fung-Chang Sung, Chi Pang Wen, Chao A. Hsiung, Chung Y. Hsu

**Affiliations:** 1 Ph.D. Program for Aging, China Medical University, Taichung, Taiwan; 2 Department of Electrical and Computer Engineering, Institute of Electrical Control Engineering, National Chiao Tung University, Hsinchu, Taiwan; 3 Institute of Population Health Sciences, National Health Research Institutes, Zhunan, Taiwan; 4 Disease Informatics Research Group, Asia University Taiwan, Taichung, Taiwan; 5 Department of Internal Medicine, Kuang Tien General Hospital, Taichung, Taiwan; 6 Institute of Population Health Sciences, National Health Research Institutes, Zhunan, Taiwan; 7 Department of Neurology, China Medical University Hospital, and School of Medicine, China Medical University, Taichung, Taiwan; 8 Brain Research Center, National Chiao Tung University, Hsinchu, Taiwan; 9 Department of Electrical and Computer Engineering, Institute of Electrical Control Engineering, National Chiao Tung University, Hsinchu, Taiwan; 10 Centre for Artificial Intelligence School of Software, Faculty of Engineering & IT, University of Technology Sydney Broadway 2007, Sydney, New South Wales, Australia; 11 Graduate Institute of Biostatistics, College of Public Health, China Medical University, Taichung, Taiwan; 12 Department of Healthcare Administration, College of Health Science, Asia University, Taichung, Taiwan; 13 Graduate Institute of Clinical Medical Science and School of Medicine, College of Medicine, China Medical University, Taichung, Taiwan; 14 Institute of Population Health Sciences, National Health Research Institutes, Zhunan, Taiwan; 15 Graduate Institute of Biomedical Science, China Medical University, Taichung, Taiwan; University of Cape Coast, GHANA

## Abstract

Exposure to air pollutants is known to have adverse effects on human health; however, little is known about the association between hydrocarbons in air and an ischemic stroke (IS) event. We investigated whether long-term exposure to airborne hydrocarbons, including volatile organic compounds, increased IS risk. This retrospective cohort study included 283,666 people aged 40 years or older in Taiwan. Cox proportional hazards regression analysis was used to fit single- and multiple-pollutant models for two targeted pollutants, total hydrocarbons (THC) and nonmethane hydrocarbons (NMHC), and estimated the risk of IS. Before controlling for multiple pollutants, hazard ratios (HRs) of IS with 95% confidence intervals for the overall population were 2.69 (2.64–2.74) at 0.16-ppm increase in THC and 1.62 (1.59–1.66) at 0.11-ppm increase in NMHC. For the multiple-pollutant models controlling for PM_2.5_, the adjusted HR was 3.64 (3.56–3.72) for THC and 2.21 (2.16–2.26) for NMHC. Our findings suggest that long-term exposure to THC and NMHC may be a risk factor for IS development.

## Introduction

Cerebrovascular diseases (CVDs) comprise conditions that lead to a cerebrovascular event, including stroke, which can be caused by a blocked artery, called ischemic stroke (IS). About 80% strokes are ISs, which occur when the arteries to the brain become narrowed or blocked, causing severely reduced blood supply and resulting in oxygen deficiency and brain cell death [1–2]. In Taiwan, stroke is the leading cause of adult disability, the third leading cause of death, and the condition associated with the second highest healthcare expenditure for adults aged ≥ 65 years [3].

Aging is the most significant factor of CVDs; however, studies have demonstrated a link between CVDs and increased ozone (O_3_) levels arising from air pollution and also a link between stroke and airborne particulate matter (PM) [4]. O_3_ is formed when hydrocarbons and nitrogen oxides (NO_X_) react with sunlight [5]. The anthropogenic emission of volatile organic compounds (VOCs), including hydrocarbons, significantly affects human health and atmospheric photochemistry [6]. Air pollutants, including methane (CH_4_), are important atmospheric precursors of O_3_ with their emission by industries and vehicles contributing to PM pollution [7]. Total hydrocarbons (THC) and nonmethane hydrocarbons (NMHC) play critical roles in the photochemical production of O_3_ and organic aerosols [8]. High O_3_ and PM levels, which are the major pollutants listed in the Pollutants Standard Index, Taiwan [9], are associated with central nervous system diseases, including IS [10]. Important sources of these pollutants include transportation (e.g., combustion and automobile engine emissions), stationary fuel (e.g., combustion from coal power plants and extraction and handling of fossil fuels), and industries (e.g., toxic chemical waste, gaseous emissions, and fuel and solvent evaporation) [11–12]. Recently, in the study using multiple-pollutant models for targeted pollutants, we have demonstrated that long-term exposure to THC and NMHC in the ambient air were associated with the risk of retinal vein obstruction [13].

The National Health Institute Research Database (NHIRD) in Taiwan, established in 1996, includes all claims data and medical histories from the National Health Insurance (NHI) database. NHIRD, which contains healthcare data of 22.96 million people (99% of Taiwan’s population) under a universal health insurance program [14], contains data on real-world practice outcomes evidences and has been increasingly recognized for its importance and clinical impact beyond results derived from multicenter clinical trials establishing the “evidence” to develop disease guidelines for clinical practice [15]. NHI has implemented strict record-keeping rules to identify medical practice misconduct or fraud and penalizes 100 times the healthcare expenditure claims to prevent such frauds. Thus, NHIRD provides high-quality healthcare data with integrity and reliability for exploring real-world evidence based on big health data analytics. The health status of each individual as recorded in the database applied the International Classification of Disease, Ninth Revision, Clinical Modification (ICD-9-CM) until recently when ICD-10-CM was implemented.

Taiwan’s sustainable energy policy, a new policy developed by the Bureau of Energy, Ministry of Economic Affairs [16], possesses a framework that aims to boost electricity generation from low-carbon emission natural gas plants and renovate coal-fired and thermal power stations relying on diesel engines. These activities will increase long-term exposure to airborne hydrocarbons in the future because the combustion of fuels produces air pollutants in the form of gases, VOCs, and PM. Many epidemiological studies have found an association between PM and increased adverse impact of human health, including IS. However, little is known about the association between hydrocarbons in air and IS event. Therefore, understanding the sources that are harmful to health can provide information for risk management strategies and could help decision makers in developing more targeted air pollution regulations. The present study aims to evaluate the adverse effect of the air pollutants THC and NMHC on human health by focusing on IS. Data from NHIRD and government environmental databases were used to examine whether long-term exposure to hydrocarbons in ambient air increased IS risk among people aged ≥ 40 years in Taiwan.

## Methods

### Data sources

Health data were obtained from the Longitudinal Health Insurance Database 2000 (LHID2000) within NHIRD, including claims data for 1 million random samples from 1996 to 2013. To enhance NHIRD data reliability, the observation period was set as 2000–2013. The Environment Resource Dataset [17] was publicly available from open government data. This dataset was obtained by the Environmental Protection Administration of Taiwan, which determined the levels of ambient pollutants and temperatures at 76 monitoring stations across Taiwan from 1993 to 2013. The Research Ethics Committee of China Medical University and Hospital in Taiwan approved the study (certificate number: CMUH-104-REC2-115-CR3). Because de-identified/anonymized data were used from NHIRD, the Research Ethics Committee did not require the right of obtaining informed consent from the patients.

### Study design and study population

**Fig 1:**
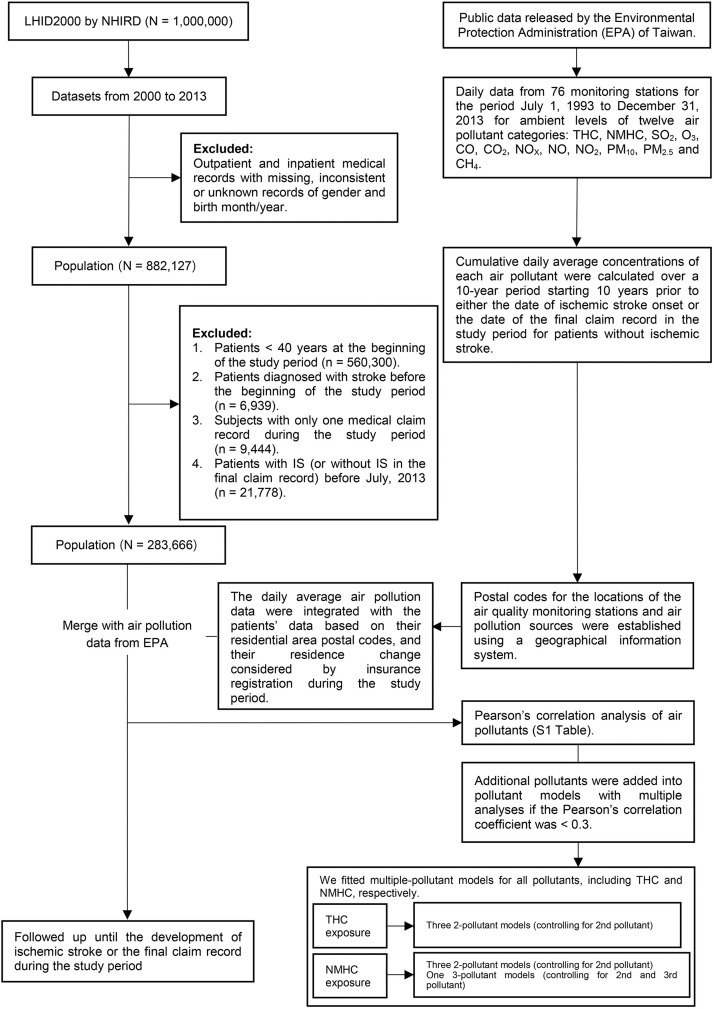
Summary of study flow.

A cohort design was used for this study from January 1, 2000 to December 31, 2013. The selection of study population is summarized in Fig 1. Among the 1 million patients in the LHID2000 database, patients aged ≥ 40 years on January 1, 2000 (n = 321,827) were included. Among them, patients with missing or unknown records for gender or birth year and month, those with stroke diagnosis (based on ICD-9-CM codes 430–438) before January 1, 2000 (n = 6,939), those with only one claim record during the study period (n = 9,444), and those with IS (or without IS in the final claim record) before July, 2013 (n = 21,778) were excluded. Finally, 283,666 patients were included in the present study.

### Selection of outcomes

From the included population, we identified people who received a first-time diagnosis of IS during the study period based on ICD-9-CM codes 433.x0, 433.x1, 434, and 436. Individuals were considered to have IS if they visited an outpatient clinic ≥ 3 times with IS diagnosis or had been hospitalized because of IS. The earliest hospitalization or visit date to an outpatient clinic with diagnosis was assigned as the diagnosis date and served as the newly diagnosed date of IS for all subsequent analyses.

### Measuring exposure to targeted pollutants

To examine the associations between newly diagnosed IS and long-term exposure to targeted air pollutants and consider the multiple pollutants effected by controlling other non-targeted pollutants over the exposure period, we determined the concentrations of 12 pollutant categories monitored by the Environmental Protection Administration in Taiwan; THC and NMHC were the study targets. The non-targeted pollutants were included in subsequent multiple-pollutant analyses. These were selected based on weak correlations (Pearson’s correlation coefficients < 0.3) of target pollutants with 10 other monitored categories: sulfur dioxide (SO_2_), O_3_, carbon monoxide (CO), carbon dioxide (CO_2_), NO_X_, nitrogen monoxide (NO), nitrogen dioxide (NO_2_), particulate matter < 10 μm in size (PM_10_), particulate matter < 2.5 μm in size (PM_2.5_), and CH_4_ (S1 Table). The daily air quality data were collected at 76 monitoring stations from July 1, 1993 to December 31, 2013 and maintained by the Environmental Protection Administration [18]. The locations where air pollutants were recorded were selected to form an integrated geographic information system. Using this system, each study patient was linked to the appropriate monitoring region by postal code, and the change of residence was considered through insurance registration during the study period. A patient’s long-term exposure to each pollutant category was defined as the cumulative concentration during the measurement period averaged per day, calculated for a 10-year period starting either 10 years before IS onset or 10 years before the final claim record date in the study period for patients without IS. Therefore, long-term exposure (LEAP) for each pollutant category (*i* = THC, NMHC, SO_2_, O_3_, CO, CO_2_, NO_X_, NO, NO_2_, PM_10_, PM_2.5_ and CH_4_) for a patient living in the region served by air quality monitoring station *j* was calculated as follows:

$$LEAP_{ij}=\frac{\sum_{t=m}^{n}AP_{ijt}}{d}$$

where *APi* is the ambient air pollution level for pollutant category, *i*, *m* is the start date of the measurement period (10 years before the IS onset date or final claim record date in the study period for patients without IS), *n* is the end date of the measurement period (IS onset date or final claim record date in the study period for patients without IS), and *d* is the number of days in the measurement period.

### Comorbidities

Information on comorbid conditions of patients was determined from LHID2000 based on ICD-9-CM codes. Comorbidities considered were hypertension (401–405); diabetes (250); hyperlipidemia (272.0–272.4); coronary heart disease (410–414); and peripheral arterial disease (440.20–440.32, 444.21–444.22, 444.81). These were identified and defined according to the diagnostic history collected from at least three outpatient visits or a single hospital admission during the 10-year period of air pollutant exposure assessment. The Charlson comorbidity index (CCI) score has been defined by Deyo et al., 1992 [19]. To prevent double-counting and over-adjustment in multivariate regressions, CCI score does not consider cerebrovascular disease as a comorbidity in patients with IS.

### Statistical analysis

The chi-squared test (for categorical variables) and one-way analysis of variance (for continuous variables) were used to test for differences in demographic characteristics and distribution of comorbidities among tertiles of the targeted pollutants concentrations. For the analyses of the time to diagnose IS, each individual’s observation time was censored at the date of the final claim record during the study period. IS risk in association with each targeted pollutant category, expressed as hazard ratios (HRs) with 95% confidence intervals (CIs), was examined using Cox proportional hazards regression, which considers potential confounders, To control the confounding effects of other pollutants, the possible link of air pollutants was used to assess the effects of multiple pollutants, by controlling other pollutants that were based on the selection of weak correlations with other air pollutants (i.e., correlation coefficients between each of the two air pollutants were lower than 0.3; S1 Table). To avoid potential collinearity problems, we did not include pollutants with high correlations in the same regression model. The effect of each targeted pollutant on the risk of newly diagnosed IS was estimated as the adjusted HR for the change of per standard deviation (SD) of 0.16 ppm for THC and 0.11 ppm for NMHC over the follow-up period.

Several studies have shown an association between air pollution exposure and neurological disorders in conjunction with the synergistic effect of temperature. The authors added temperature in the particulate air pollution model to control the effect of weather conditions on air pollution and stroke mortality [20] because both cold and hot temperatures were associated with increased risk of stroke mortality [21]. Elevated mortality due to cardiovascular diseases has been shown to be related to extreme temperature; the increase and decrease in ambient temperature has a relationship with cardiovascular mortality [22–23]. Therefore, to control the effects of weather conditions on air pollution and dementia, ambient temperature would be one of the adjusting factors in the pollutant models. Additionally, to control the short-term pollutant exposure effects, we used a lag of 0–2 days (average of the concentration levels on the same day of IS onset, one day, and two days before) for all air pollutants [24] as one of the adjusting factors. Because air pollutant levels vary depending on the weather conditions, adjustment for the season is usually considered a vital modifier in ambient air pollution-related biological effects [25]. In the present study, multiple-pollutant models for two targeted pollutants were fitted, the independent effects of each targeted pollutant by adjusting for age, gender, insurance amount, CCI score, hypertension, diabetes, hyperlipidemia, coronary heart disease, peripheral arterial disease, lag of 0–2 days, season (seasonal trends in IS onset), and ambient temperature were estimated, and other pollutants that showed weak correlations were controlled. The data from the concentration of each targeted pollutant category were divided into three levels using tertiles, and adjusted HRs with 95% CIs were re-calculated.

Stratified analyses examined whether the effects of the pollutant categories differed between males and females. Plots of Kaplan–Meier analysis determined the probability of people remaining with IS, and the log-rank test evaluated the difference among tertiles of concentrations of pollutant categories. The analyses were performed using CareStore X1 Studio Research Platform and the Statistical Product and Service Solutions (SPSS; Version 22). All statistical tests were two-sided; *p* values of 0.05 were considered to be statistically significant.

## Results

### Study population characteristics

The demographic data and comorbid states among tertiles of targeted pollutant categories are presented in Tables 1 and 2, with T1 and T3 being the lowest and highest levels, respectively. The mean age for initiating analysis was 53.4 ± 10.1, 54.1 ± 11.0, and 56.2 ± 12.5 years at T1, T2, and T3 levels for THC, respectively. Moreover, the mean age of initiating analysis was 55.0 ± 11.0, 55.0 ± 11.7, and 53.5 ± 11.2 years at T1, T2, and T3 levels for NMHC, respectively. The patients included in this study had a mean age of 54.5 ± 11.3 years. In both THC and NMHC, the patients were slightly older in the highest level of the tertile (T3) and more frequently exhibited comorbidities at T3 for THC compared with other tertiles.

**Table 1:** Characteristics of the study population among tertiles of total hydrocarbons (THC) exposure.

Characteristics	Tertiles of average daily THC^*a*^, n (%)			*P* value^*b*^	Total (N = 279,398)
	T1 (lowest) (n = 93,126)	T2 (n = 96,097)	T3 (highest) (n = 90,175)		
**Ischemic stroke**	1,499 (1.6)	4,670 (4.9)	10,698 (11.9)	<0.001	16,867 (6.1)
**Age**, years				<0.001	
Mean ± SD	53.4 ± 10.1	54.1 ± 11.0	56.2 ± 12.5		54.5 ± 11.3
**Gender**				<0.001	
Male	44,160 (47.4)	48,256 (50.2)	49,328 (54.7)		141,744 (50.8)
**Insurance amount**^*c*^, NT$				<0.001	
<19,999	19,074 (20.5)	22,108 (23.0)	28,602 (31.7)		69,784 (25.0)
20,000−39,999	4,306 (4.6)	5,509 (5.7)	6,477 (7.2)		16,292 (5.8)
≥40,000	60,315 (64.8)	63,279 (65.9)	50,353 (55.8)		173,947 (62.4)
unknown	9,431 (10.1)	5,201 (5.4)	4,743 (5.3)		19,375 (6.8)
**CCI score**				<0.001	
Mean ± SD	1.2 ± 1.7	1.4 ± 2.0	1.9 ± 2.5		1.5 ± 2.1
**Comorbidities**^*d*^					
Hypertension	20,358 (21.9)	19,830 (20.6)	18,510 (20.5)	<0.001	58,698 (21.0)
Diabetes	14,535 (15.6)	15,371 (16.0)	18,440 (20.4)	<0.001	48,346 (17.3)
Hyperlipidemia	21,079 (22.6)	21,448 (22.3)	19,036 (21.1)	<0.001	61,563 (22.0)
Coronary heart disease	13,034 (14.0)	14,795 (15.4)	17,497 (19.4)	<0.001	45,326 (16.3)
Peripheral arterial disease	1,136 (1.2)	1,307 (1.4)	1,344 (1.5)	<0.001	3,787 (1.4)

SD, standard deviation; CCI score, Charlson Comorbidity Index score.

^*a*^The tertile values, in ppm, are as follows: T1: < 2.18, T2: ≥ 2.18 and < 2.33, and T3: ≥ 2.33.

^*b*^The chi-squared test or one-way analysis of variance among tertiles of total hydrocarbons.

^*c*^Insurance amount was measured as the average value during 10-year period of air pollutant exposure assessment.

^*d*^Comorbidities were during 10-year period of air pollutant exposure assessment.

**Table 2:** Characteristics of the study population among tertiles of nonmethane hydrocarbons (NMHC) exposure.

Characteristics	Tertiles of average daily NMHC^*a*^, n (%)			*P* value^*b*^	Total (N = 279,398)
	T1 (lowest) (n = 93,610)	T2 (n = 93,432)	T3 (highest) (n = 92,356)		
**Ischemic stroke**	4,088 (4.4)	6,199 (6.6)	6,580 (7.1)	<0.001	16,867 (6.1)
**Age**, years				<0.001	
Mean ± SD	55.0 ± 11.0	55.0 ± 11.7	53.5 ± 11.2		54.5 ± 11.3
**Gender**				<0.001	
Male	46,331 (49.5)	46,144 (49.4)	49,269 (53.3)		141,744 (50.8)
**Insurance amount**^*c*^, NT$				<0.001	
<19,999	16,428 (17.5)	22,962 (24.6)	30,394 (32.9)		69,784 (25.0)
20,000–39,999	3,798 (4.1)	5,289 (5.7)	7,205 (7.8)		16,292 (5.8)
≥40,000	69,116 (73.8)	51,624 (55.3)	53,207 (57.6)		173,947 (62.4)
unknown	4,268 (4.6)	13,557 (14.5)	1,550 (1.7)		19,375 (6.8)
**CCI score**				<0.001	
Mean ± SD	1.4 ± 2.0	1.6 ± 2.2	1.4 ± 2.1		1.5 ± 2.1
**Comorbidities**^*d*^					
Hypertension	20,312 (21.7)	19,773 (21.2)	18,613 (20.2)	<0.001	58,698 (21.0)
Diabetes	15,916 (17.0)	16,504 (17.7)	15,926 (17.2)	0.001	48,346 (17.3)
Hyperlipidemia	20,742 (22.2)	20,144 (21.6)	20,677 (22.4)	<0.001	61,563 (22.0)
Coronary heart disease	14,915 (15.9)	15,727 (16.8)	14,684 (15.9)	<0.001	45,326 (16.3)
Peripheral arterial disease	1,300 (1.4)	1,337 (1.4)	1,150 (1.2)	0.001	3,787 (1.4)

SD, standard deviation; CCI score, Charlson Comorbidity Index score.

^*a*^The tertile values, in ppm, are as follows: T1: < 0.25, T2: ≥ 0.25 and < 0.33, and T3: ≥ 0.33.

^*b*^The chi-squared test or one-way analysis of variance among tertiles of nonmethane hydrocarbons.

^*c*^Insurance amount was measured as the average value during 10-year period of air pollutant exposure assessment.

^*d*^Comorbidities were during 10-year period of air pollutant exposure assessment.

### Air pollution

The mean of daily average of THC concentration was 2.27 ppm (SD = 0.16), whereas; the mean of daily average of NMHC was 0.30 ppm (SD = 0.11) over the 10-year exposure period in the current study. The summary statistics of the air pollutants are shown in S2 Table. The distributions of daily average concentrations of air pollutants over the 10-year exposure period are shown in S1 and S2 Figs. The concentration of THC slightly correlated with SO_2_ (r = 0.129), PM_10_ (r = −0.156), and PM_2.5_ (r = −0.162), whereas the concentration of NMHC slightly correlated with SO_2_ (r = 0.186), PM_2.5_ (r = −0.254), and CH_4_ (r = 0.253) (S1 Table).

### Associations between IS and pollutant categories

**Fig 2:**
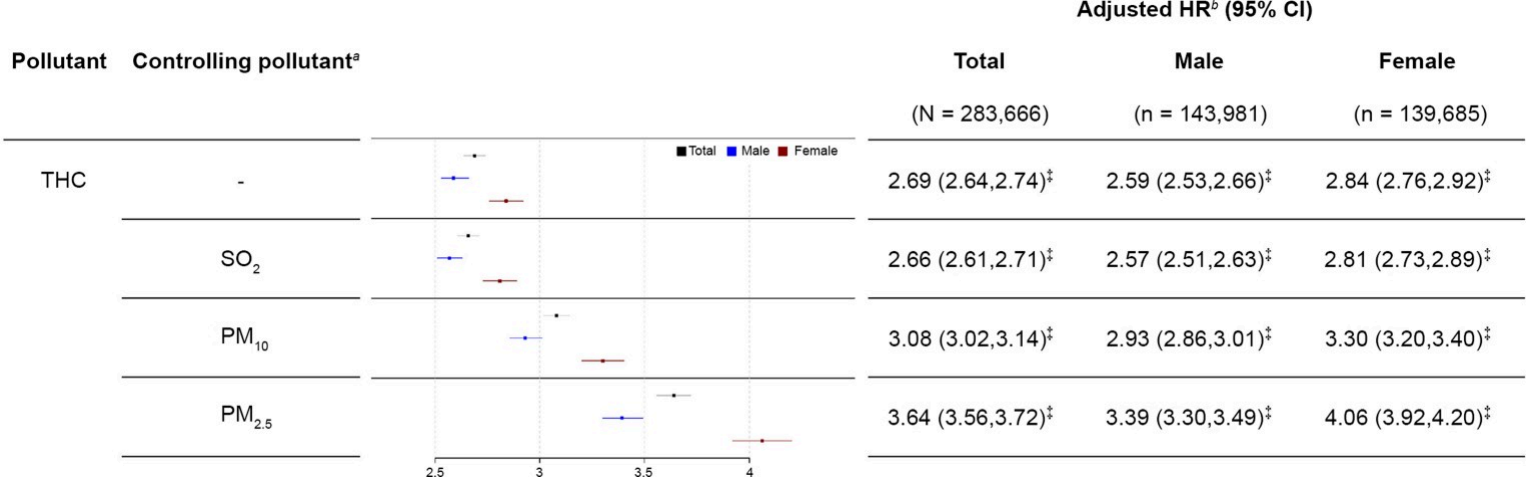
Forest plot of long-term THC exposure at a 0.16-ppm increment associated with the incidence of ischemic stroke. HR, hazard ratio; CI, confidence interval; SO_2_, sulfur dioxide; PM_10_, particulate matter < 10 μm in size; PM_2.5_, particulate matter < 2.5 μm in size. ^*a*^Additional pollutants were added into THC models for multiple analysis only when Pearson’s correlation coefficient was < 0.3. ^*b*^Cox regression models were adjusted for age, gender, insurance amount, CCI score, hypertension, diabetes, hyperlipidemia, coronary heart disease, peripheral arterial disease, lag0-2, season, and ambient temperature, controlled pollutants (weak correlation with THC). ^‡^*p* < 0.001.

Fig 2 presents the single- and multiple-pollutant models for per SD of 0.16 ppm increase in THC. Before controlling for other pollutants, newly diagnosed IS was positively associated with the daily average concentration over the 10-year period for THC with adjusted HR of 2.69 (95% CI: 2.64–2.74, *p* < 0.001), indicating that an increase in THC by 0.16 ppm increased the likelihood of having IS by 169%. The appropriateness of the Cox proportional hazards model is supported by the plot in S3 Fig, where the log (−log (survival function)) versus log of survival time is plotted for the tertiles of THC. Adjusted HR (95% CI) of IS was 2.59 (2.53–2.66, *p* < 0.001) for males and 2.84 (2.76–2.92, *p* < 0.001) for females. Fig 2 shows the changes in adjusted HRs for different multiple-pollutant models. We fitted two-pollutant models for THC with controlling for the concomitant exposure to SO_2_, PM_10,_ or PM_2.5_. Among them, controlling for PM_2.5_ resulted in adjusted HRs for all patients, males, and females of 3.64 (3.56–3.72), 3.39 (3.30–3.49), and 4.06 (3.92–4.20), respectively. In our findings, we observed slight changes in the effects of THC after controlling for SO_2_, PM_10,_ or PM_2.5_, and the directions of the effect estimates did not change, suggesting that our findings were robust against this potential confounder.

**Fig 3:**
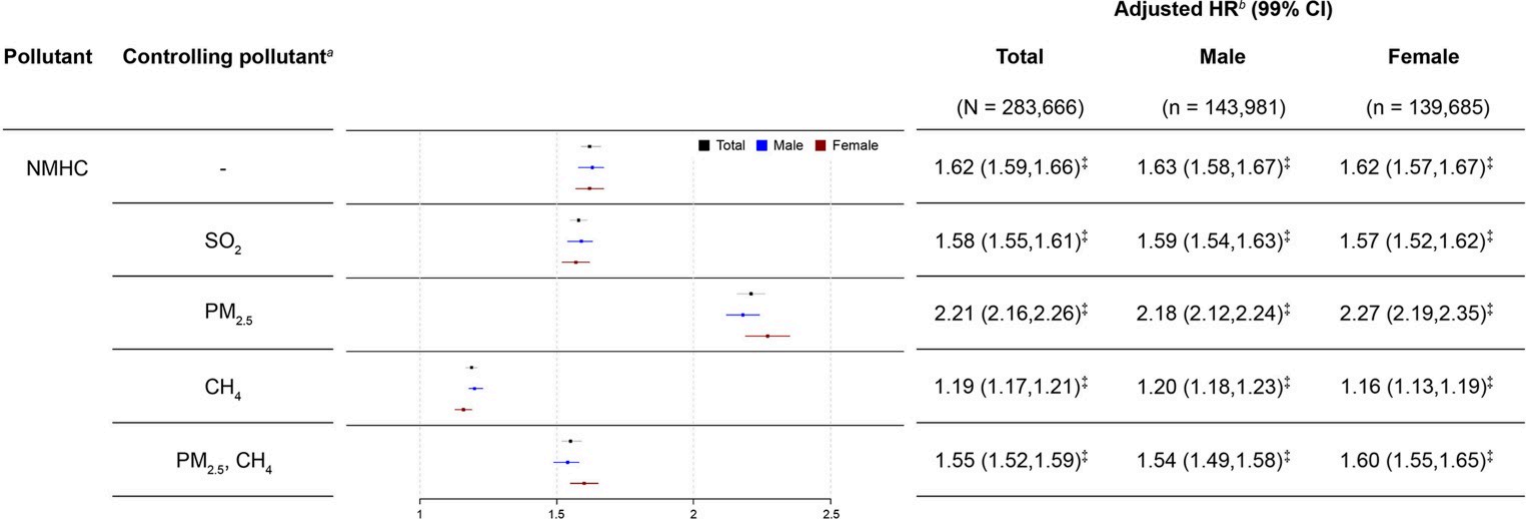
Forest plot of long-term exposure to NMHC at a 0.11-ppm increment associated with the incidence of ischemic stroke. HR, hazard ratio; CI, confidence interval; SO_2_, sulfur dioxide; CH_4_, methane; PM_2.5_, particulate matter < 2.5 μm in size. ^*a*^Additional pollutants were added into NMHC models for multiple analysis only when Pearson’s correlation coefficient was < 0.3. ^*b*^Cox regression models were adjusted for age, gender, insurance amount, CCI score, hypertension, diabetes, hyperlipidemia, coronary heart disease, peripheral arterial disease, lag0-2, season, and ambient temperature, controlled pollutants (weak correlation with NMHC). ^‡^*p* < 0.001.

Fig 3 presents the single- and multiple-pollutant models for per SD of 0.11 ppm increase in NMHC. Before controlling for multiple pollutants, adjusted HRs (95% CIs) of IS for the overall population, males, and females were 1.62 (1.59–1.66), 1.63 (1.58–1.67), and 1.62 (1.57–1.67), respectively. We fitted two-pollutant models for NMHC with controlling for the concomitant exposure to SO_2_, PM_2.5,_ or CH_4_ and the three-pollutant model for NMHC with controlling for PM_2.5_ and CH_4_. Controlling for PM_2.5_ gave the adjusted HRs (95% CIs) of 2.21 (2.16–2.26), 2.18 (2.12–2.24), and 2.27 (2.19–2.35), respectively. The slightly changed effects and no change in directions of the effects of NMHC were observed after controlling for other pollutants. The appropriateness of the Cox proportional hazards model is supported by the plot in S4 Fig, where the log (−log (survival function)) versus log of survival time is plotted for the tertiles of NMHC.

Table 3 presents the Cox proportional hazards regression analysis of the two targeted pollutant categories divided into three levels by the tertiles. In each case, the lowest tertile was used as the reference, and the estimated HRs were adjusted for age, gender, insurance amount, lag0-2, season, ambient temperature, and comorbidities. These results are consistent with those obtained from the earlier multivariate analyses.

**Table 3:** Association between air pollutants divided into the tertiles and IS risk.

Pollutant category	Tertiles of average daily pollutant^*a*^	Population	IS	PY	Adjusted HR^*b*^ (95% CI)
THC	T1 (lowest)	Total (N = 283,666)	1,499	1,228,402	1.00 (reference)
	T2		4,670	1,194,359	1.98 (1.86,2.12)^**‡**^
	T3 (highest)		10,698	908,454	7.64 (7.15,8.16)^**‡**^
	*p* for trend				< 0.001
	T1 (lowest)	Male (n = 143,981)	43,268	892	1.00 (reference)
	T2		45,784	2,472	1.71 (1.57,1.86)^**‡**^
	T3 (highest)		43,294	6,034	6.48 (5.95,7.06)^**‡**^
	*p* for trend				< 0.001
	T1 (lowest)	Female (n = 139,685)	48,359	607	1.00 (reference)
	T2		45,643	2,198	2.37 (2.14,2.62)^**‡**^
	T3(highest)		36,183	4,664	9.28 (8.38,10.29)^**‡**^
	*p* for trend				< 0.001
NMHC	T1 (lowest)	Total (N = 283,666)	4,088	1,172,107	1.00 (reference)
	T2		6,199	1,107,719	1.18 (1.12,1.25)^**‡**^
	T3 (highest)		6,580	1,051,390	1.29 (1.22,1.37)^**‡**^
	*p* for trend				< 0.001
	T1 (lowest)	Male (n = 143,981)	44,097	2,234	1.00 (reference)
	T2		42,918	3,226	1.23 (1.14,1.31)^**‡**^
	T3 (highest)		45,331	3,938	1.33 (1.23,1.43)^**‡**^
	*p* for trend				< 0.001
	T1 (lowest)	Female (n = 139,685)	45,425	1,854	1.00 (reference)
	T2		44,315	2,973	1.11 (1.03,1.21)^**†**^
	T3 (highest)		40,445	2,642	1.24 (1.13,1.36)^**‡**^
	*p* for trend				< 0.001

IS, ischemic stroke; PY, person years; HR, hazard ratio; CI, confidence interval; THC, total hydrocarbons; NMHC, nonmethane hydrocarbons.

^*a*^The tertile values, in ppm (THC, NMHC), are as follows:

THC (T1: < 2.18, T2: ≥ 2.18 and < 2.33, and T3: ≥ 2.33); NMHC (T1: < 0.25, T2: ≥ 0.25 and < 0.33, and T3: ≥ 0.33).

^*b*^Cox regression models were adjusted for age, gender, insurance amount, CCI score, hypertension, diabetes, hyperlipidemia, coronary heart disease, peripheral arterial disease, lag0-2, season, and ambient temperature.

^**†**^*p* < 0.01,

^‡^*p* < 0.001.

For those exposed to the highest tertile (T3) of THC for the overall population, the adjusted HR (95% CI) of IS was 7.64 (7.15–8.16, *p* < 0.001), indicating that those exposed to average daily levels of ≥ 2.33 ppm THC were 664% more likely to have newly diagnosed IS than those exposed to < 2.18 ppm THC (values corresponding to the tertiles are given in the note of Table 3). Among the various THC concentrations to which all patients were exposed, THC levels were categorized into tertiles, T1 being the lowest and T3 being the highest. The adjusted HRs (95% CIs) of IS for T3 were 6.48 (5.95–7.06, *p* < 0.001) for males and 9.28 (8.38–10.29, *p* < 0.001) for females.

For NMHC, the adjusted HRs (95% CIs) of IS for T3 exposure relative to T1 exposure for the overall population, males, and females were 1.29 (1.22–1.37), 1.33 (1.23–1.43), and 1.24 (1.13–1.36), respectively. When data on gender were stratified or merged for analysis, statistically significant correlations of adjusted HRs were measured for T1 compared with T2 and T3. The analysis demonstrated an association between the two targeted pollutants and IS risk.

Cumulative IS incidence for the two targeted pollutants was assessed using the Kaplan–Meier method (S5 Fig), presenting a clear trend of increased IS risk with increased each targeted pollutant exposure. Statistically significant differences in IS occurrence were observed among the tertiles of the targeted pollutant categories (log-rank test, *p* < 0.001).

## Discussion

This population-based cohort study linked national insurance claims data to open government data to investigate the association between long-term exposure to selected air pollutants in Taiwan and the IS risk. An adverse impact of VOCs in the ambient air on IS risk was observed in individuals aged ≥ 40 years with average daily exposure over a 10-year period. The major findings of this study were that increased exposure to airborne hydrocarbons (THC or NMHC) is associated with an enhanced risk of IS for overall population, males and females. The results collectively show that increasing levels of the two targeted pollutants increases the IS risk in a dose-dependent manner (Figs 2 and 3).

To control for potential confounding effects from other pollutants, we further evaluated whether the associations were changed by concomitant exposure to other air pollutants by using a multiple-pollutant model for analysis. We found that the associations in our study remained after controlling for simultaneous exposure to other pollutants. Previous studies have suggested that short-term changes in ambient PM_2.5_ levels were associated with increased risk of IS onset [26], and long-term exposure to higher levels of PM_2.5_ have been associated with a higher risk of stroke [27]. After evaluating the modification effects of PM_2.5_ on the associations of targeted pollutants and IS, two-pollutant models showed that PM_2.5_ had a noticeable impact on the associations of IS with THC and NMHC, and the effects increased after adjustment for PM_2.5_. Therefore, the effects of THC and NMHC on IS in single-pollutant models were reliable.

In the current study, we also used single-pollutant models to evaluate the association between other pollutants (such as PM_2.5_, NO_X_, and O_3_) and IS incidence, which demonstrated that the effects of air pollutant exposure on IS were reliable. After adjusting for confounding factors, the adjusted HRs (95% CIs) were 1.51 (1.48–1.55) for PM_2.5_, 1.67 (1.63–1.71) for NO_X_, and 0.47 (0.46–0.48) for O_3_. We observed the same directions of risk estimates, associating exposure to PM_2.5_ with IS incidence, as in previous studies [26–28]; short-term exposure to NO_X_ with first-time IS admissions in Copenhagen [29]; and short-term exposure to O_3_ with IS incidence and admissions [26,30]. The estimates for these associations were slightly high and remained statistically significant. Earlier studies suggested that the risk of stroke and CVD per unit concentration associated with long-term exposure to air pollution was substantially higher than the risk associated with acute exposures [27], and this could be related to the accumulated effect of prolonged exposure. Additionally, low environmental O_3_ exposure might be more beneficial against IS onset [31] and could exert neuroprotective effects by regulating the inflammatory response, improving cerebrovascular rheology, and strengthening antioxidant response in hypoxic brains [32]. Several epidemiological studies examined the association of O_3_ with IS but reported inconsistent results. Some studies reported positive associations [33], whereas others presented no or negative associations [34–35].

The potential mechanism underlying the associations found in the current study is the inflammatory processes in the brain. One study proposed that inflammation plays an important role in the pathogenesis of IS and other forms of ischemic brain injury [36]. Several studies have demonstrated a relationship between hydrocarbons and inflammatory response. Environmental exposure to polycyclic aromatic hydrocarbons (PAHs) is prevalent and adversely impacts health. Exposure to PAHs is associated with oxidative stress and inflammatory response [37]. In another study, the authors found that PAH exposure was associated with levels of putative cardiovascular disease-related proteins in serum. The findings show that differentially expressed proteins are mainly involved in inflammatory response and immunological functions, such as leukocyte migration, leukocyte cell movement, and immune cell adhesion [38]. In addition, some PAHs metabolites also show consistent positive association with C-reactive protein, a plasma inflammation marker [39].

Many studies have demonstrated that air pollution adversely impacts health, including IS; however, few have concentrated on hydrocarbons in ambient air. VOCs in diesel engine exhaust emissions, including benzene and formaldehyde. They can be positively correlated with THC emissions, contributing to greenhouse effect and global warming by depleting the ozone layer [12]. Long-term exposure to traffic-related air pollution increases the risk of cardiopulmonary and lung cancer mortalities and provides additional evidence for adverse effects on intracerebral hemorrhage and IS [40]. Air pollution is a multifaceted environmental toxicant comprising a diverse mixture of PM and gases. Gaseous pollutants could be the predictors of acute IS mortality [41], and short-term exposure to PM_10_ and SO_2_ is associated with IS caused by cardioembolism [42]. The speculative mechanism linking air pollution to cardiovascular disease and increased IS risk include systemic inflammatory responses, systemic oxidative stress, vascular endothelial cell injury, a prothrombotic state, acute arterial vasoconstriction, and atherosclerotic progression [43].

Chronic exposure to air pollution may have a direct effect on the brain by triggering and/or promoting neurodegeneration or by inducing clinically silent ischemic lesions; for example, due to slowly progressing athero-thrombosis, which leads to lacunar stroke. Air pollution could be associated with the risk of stroke due to its direct or indirect effects or a combination of the two. Direct damage can be caused through inflammation and neurodegeneration, causing Alzheimer's disease (AD), dementia, or stroke [44]. A previous study has examined the effect of chronic exposure to air pollution on the biomarkers of AD in brain tissues of postmortem humans or on experimental animals [44]. Inflammation entails multiple cellular, hormonal, and biochemical alterations that are systemic and organ specific. Various acute and chronic infections and many exogenous and intrinsic sources of inflammation are associated with increased IS risk. Furthermore, systemic infections with resultant immune or inflammatory processes are related to stroke etiology and pathology. Therefore, chronic inflammation plays a role in atherothrombotic diseases, including stroke, and has therefore been added to the list of established risk factors for stroke [45].

The strengths of this study are the following. First, this is a nationwide study conducted using a large population derived from NHIRD, which contains healthcare data of 22.96 million people (99% of Taiwan’s population) under a universal health insurance program. Second, this study is based on a long term of a 10-year follow-up period, which allows the possible occurrence of IS to be assessed. Third, little is known about the epidemiological studies that evaluate the association between hydrocarbons in air and IS event. We have considered and assessed the association between possible synergistic effect on multiple air pollutants for airborne hydrocarbons and IS risk. With the global ageing trend, the prevalence and burden of IS are also likely to expand. Sources that are harmful to health could provide the information for risk management strategies and help decision makers in developing more targeted air pollution regulations. These findings may have significant public health implications for the prevention of IS.

There were several limitations to this study. First, potential biases resulting from unknown confounders, associated with the confounding factors for which adjustments were made, may have affected the results. We were unable to adjust for confounders, including genetic information and relevant clinical variables (e.g., imaging results, physiologic levels), because of the unavailability of relevant information in NHIRD. Except for age, gender, and insurance amount, we considered six risk factors for potential risk factors for IS. Because of the paucity of data sources, the effects of other clinical factors, including blood pressure levels, total cholesterol, and eGFR, were not assessed in the current study [46–48]. In addition, humidity levels have also been evidenced as a triggering factor for stroke [49] and without it as a confounding factor because of the lack of data in the Environment Resource Dataset. Second, although our exposure assessment relied on residences and accounted for each patient's registration of moved during the study, this does not completely reflect personal exposure. To protect patient privacy, NHIRD does not provide patient addresses, workplaces and types. Therefore, we used the participants’ insurance registration during the exposure period to assign them a residential district by the postal code. This could have led to misclassification of exposure, adversely affecting the accuracy of the study results.

## Conclusions

This retrospective cohort study offered new evidence that long-term exposure to THC and NMHC may be a risk factor for IS. The results indicate the possible link between air pollutants, including THC and NMHC, and IS risk. Further, long-term exposure to increased levels of both targeted pollutant categories is associated with an increased IS risk in stratified analyses by gender in the Taiwanese population.

## Supporting information

S1 Table Pearson’s correlation analysis for air pollutants over 10-year exposure period. CO_2_, carbon dioxide; CO, carbon monoxide; CH_4,_ methane; NMHC, nonmethane hydrocarbons; NO, nitrogen monoxide; NO_2,_ nitrogen dioxide; NO_X_, nitrogen oxides; O_3_, ozone; PM_10_, particulate matter < 10 μm in size; PM_2.5,_ particulate matter < 2.5 μm in size; SO_2_, sulfur dioxide; THC, total hydrocarbons. ^†^Correlation significant at the 0.01 level (two-tailed). *Correlation significant at the 0.05 level (two-tailed). Correlation coefficient values of < 0.3 denote a low strength of correlation, which qualified as the controlling pollutant in multiple-pollutant models of targeted pollutants.
(DOCX)

S2 Table Mean and distribution of air pollutants over 10-year exposure period. SD, standard deviation; 5th, 5 percentile; 95th, 95 percentile; Min, minimum; Max, maximum; IQR, interquartile range; T_1_, 33.33 percentile; T_2_, 66.66 percentile; ppb, parts per billion; ppm, parts per million; μg/m^3^, microgram/cubic meter; CO_2_, carbon dioxide; CO, carbon monoxide; CH_4,_ methane; NMHC, nonmethane hydrocarbons; NO, nitrogen monoxide; NO_2,_ nitrogen dioxide; NO_X_, nitrogen oxides; O_3_, ozone; PM_10_, particulate matter < 10 μm in size; PM_2.5,_ particulate matter < 2.5 μm in size; SO_2_, sulfur dioxide; THC, total hydrocarbons.
(DOCX)

S1 Fig Distributions of the daily average concentrations of air pollutants (SO_2_, CO_2_, CO, O_3_, PM_10_, and PM_2.5_) over 10-year exposure period. (DOCX)

S2 Fig Distributions of the daily average concentrations of air pollutants (NO_X_, NO, NO_2_, THC, NMHC, and CH_4_) over 10-year exposure period. (DOCX)

S3 Fig The plot of log (−log (survival function)) versus log of survival time in THC. The tertile values, in ppm, are as follows: T1: < 2.18, T2: ≥ 2.18 and < 2.33, and T3: ≥ 2.33.
(DOCX)

S4 Fig The plot of log (−log (survival function)) versus log of survival time in NMHC. The tertile values, in ppm, are as follows: T1: < 0.25, T2: ≥ 0.25 and < 0.33, and T3: ≥ 0.33.
(DOCX)

S5 Fig **Cumulative incidence of ischemic stroke for individuals among tertiles of pollutant categories: (A) THC and (B) NMHC**. The tertile values, in ppm (THC, NMHC), are as follows: THC (T1: < 2.18, T2: ≥ 2.18 and < 2.33, and T3: ≥ 2.33); NMHC (T1: < 0.25, T2: ≥ 0.25 and < 0.33, and T3: ≥ 0.33).
(DOCX)
