# Supplementary material for: Long-term ambient hydrocarbons exposure and incidence of ischemic stroke
Source: PLoS One. 2019 Dec 4;14(12):e0225363. doi: 10.1371/journal.pone.0225363 (PMC6892494; doi:10.1371/journal.pone.0225363)
Supplement: S1 Table — CO2, carbon dioxide; CO, carbon monoxide; CH4, methane; NMHC, nonmethane hydrocarbons; NO, nitrogen monoxide; NO2, nitrogen dioxide; NOX, nitrogen oxides; O3, ozone; PM10, particulate matter < 10 μm in size; PM2.5, particulate matter < 2.5 μm in size; SO2, sulfur dioxide; THC, total hydrocarbons. †Correlation significant at the 0.01 level (two-tailed). *Correlation significant at the 0.05 level (two-tailed). Correlation coefficient values of < 0.3 denote a low strength of correlation, which qualified as the controlling pollutant in multiple-pollutant models of targeted pollutants. (DOCX) [file pone.0225363.s001.docx]

# **Supporting Information**

# **Long-term ambient hydrocarbons exposure and incidence of ischemic stroke**

**S1 Table. Pearson’s correlation analysis for air pollutants over 10-year exposure period**

|  | SO_2_ | CO_2_ | CO | O_3_ | PM_10_ | PM_2.5_ | NO_X_ | NO | NO_2_ | THC | NMHC | CH_4_ |
| --- | --- | --- | --- | --- | --- | --- | --- | --- | --- | --- | --- | --- |
| SO_2_ | 1 | **0.110^†^** | **0.229^†^** | **0.016^†^** | 0.613^†^ | 0.618^†^ | 0.313^†^ | **0.120^†^** | 0.486^†^ | **0.129^†^** | **0.186^†^** | **0.007^†^** |
| CO_2_ |  | 1 | -0.359^†^ | **0.102^†^** | 0.440^†^ | **0.284^†^** | **-0.243^†^** | -0.326^†^ | **-0.134^†^** | -0.534^†^ | -0.457^†^ | -0.421^†^ |
| CO |  |  | 1 | -0.612^†^ | **-0.236^†^** | **-0.138^†^** | 0.948^†^ | 0.927^†^ | 0.870^†^ | 0.736^†^ | 0.901^†^ | **0.239^†^** |
| O_3_ |  |  |  | 1 | 0.304^†^ | **0.264^†^** | -0.531^†^ | -0.478^†^ | -0.532^†^ | -0.441^†^ | -0.464^†^ | **-0.230^†^** |
| PM_10_ |  |  |  |  | 1 | 0.927^†^ | **-0.197^†^** | -0.378^†^ | **0.019^†^** | **-0.156^†^** | -0.309^†^ | **0.071^†^** |
| PM_2.5_ |  |  |  |  |  | 1 | **-0.124^†^** | -0.314^†^ | **0.095^†^** | **-0.162^†^** | **-0.254^†^** | **0.004^*^** |
| NO_X_ |  |  |  |  |  |  | 1 | 0.952^†^ | 0.945^†^ | 0.692^†^ | 0.882^†^ | **0.186^†^** |
| NO |  |  |  |  |  |  |  | 1 | 0.799^†^ | 0.756^†^ | 0.922^†^ | **0.248^†^** |
| NO_2_ |  |  |  |  |  |  |  |  | 1 | 0.550^†^ | 0.743^†^ | **0.099^†^** |
| THC |  |  |  |  |  |  |  |  |  | 1 | 0.809^†^ | 0.772^†^ |
| NMHC |  |  |  |  |  |  |  |  |  |  | 1 | **0.253^†^** |
| CH_4_ |  |  |  |  |  |  |  |  |  |  |  | 1 |

CO_2_, carbon dioxide; CO, carbon monoxide; CH_4,_ methane; NMHC, nonmethane hydrocarbons; NO, nitrogen monoxide; NO_2,_ nitrogen dioxide; NO_X_, nitrogen oxides; O_3_, ozone; PM_10_, particulate matter < 10 μm in size; PM_2.5,_ particulate matter < 2.5 μm in size; SO_2_, sulfur dioxide; THC, total hydrocarbons.

^†^Correlation significant at the 0.01 level (two-tailed).

^*^Correlation significant at the 0.05 level (two-tailed).

**Correlation coefficient values of <0.3 denote a low strength of correlation, which qualified as the controlling pollutant in multiple-pollutant models of targeted pollutants.**
