# Supplementary material for: Long-term ambient hydrocarbons exposure and incidence of ischemic stroke
Source: PLoS One. 2019 Dec 4;14(12):e0225363. doi: 10.1371/journal.pone.0225363 (PMC6892494; doi:10.1371/journal.pone.0225363)
Supplement: S2 Table — SD, standard deviation; 5th, 5 percentile; 95th, 95 percentile; Min, minimum; Max, maximum; IQR, interquartile range; T1, 33.33 percentile; T2, 66.66 percentile; ppb, parts per billion; ppm, parts per million; μg/m3, microgram/cubic meter; CO2, carbon dioxide; CO, carbon monoxide; CH4, methane; NMHC, nonmethane hydrocarbons; NO, nitrogen monoxide; NO2, nitrogen dioxide; NOX, nitrogen oxides; O3, ozone; PM10, particulate matter < 10 μm in size; PM2.5, particulate matter < 2.5 μm in size; SO2, sulfur dioxide; THC, total hydrocarbons. (DOCX) [file pone.0225363.s002.docx]

**S2 Table. Mean and distribution of air pollutants over 10-year exposure period**

|  | Mean | SD | Median | 5^th^ | 95th | Min | Max | IQR | T_1_ | T_2_ |
| --- | --- | --- | --- | --- | --- | --- | --- | --- | --- | --- |
| SO_2_ (ppb) | 4.21 | 1.41 | 3.87 | 2.52 | 7.66 | 0.50 | 11.25 | 0.71 | 3.56 | 4.04 |
| CO_2_ (ppm) | 400.72 | 10.57 | 398.93 | 385.36 | 418.07 | 343.49 | 461.64 | 9.82 | 397.50 | 403.20 |
| CO (ppm) | 0.55 | 0.14 | 0.53 | 0.37 | 0.75 | 0.25 | 1.32 | 0.20 | 0.47 | 0.62 |
| O_3_ (ppb) | 28.31 | 2.37 | 27.88 | 24.50 | 32.29 | 1.03 | 52.20 | 2.19 | 27.44 | 28.77 |
| PM_10_ (μg/m^3^) | 55.20 | 10.34 | 54.66 | 38.77 | 71.10 | 1.04 | 85.20 | 15.33 | 47.36 | 58.46 |
| PM_2.5_ (μg/m^3^) | 33.16 | 7.14 | 33.71 | 21.86 | 45.61 | 1.00 | 101.00 | 9.54 | 27.68 | 35.99 |
| NO_X_ (ppb) | 25.97 | 8.24 | 25.01 | 14.15 | 37.17 | 1.03 | 58.53 | 13.81 | 20.60 | 30.79 |
| NO (ppb) | 7.66 | 4.50 | 6.19 | 2.86 | 15.10 | 0.12 | 30.26 | 7.36 | 4.73 | 7.98 |
| NO_2_ (ppb) | 18.31 | 4.19 | 18.78 | 10.65 | 23.50 | 1.02 | 28.65 | 6.97 | 16.05 | 21.36 |
| THC (ppm) | 2.27 | 0.16 | 2.26 | 2.03 | 2.52 | 1.00 | 3.45 | 0.20 | 2.18 | 2.33 |
| NMHC (ppm) | 0.30 | 0.11 | 0.28 | 0.15 | 0.47 | 0.06 | 1.15 | 0.11 | 0.25 | 0.33 |
| CH_4_ (ppm) | 1.98 | 0.10 | 1.97 | 1.82 | 2.16 | 1.00 | 2.83 | 0.09 | 1.95 | 2.00 |

SD, standard deviation; 5th, 5 percentile; 95th, 95 percentile; Min, minimum; Max, maximum; IQR, interquartile range; T_1_, 33.33 percentile; T_2_, 66.66 percentile; ppb, parts per billion; ppm, parts per million; μg/m^3^, microgram/cubic meter; CO_2_, carbon dioxide; CO, carbon monoxide; CH_4,_ methane; NMHC, nonmethane hydrocarbons; NO, nitrogen monoxide; NO_2,_ nitrogen dioxide; NO_X_, nitrogen oxides; O_3_, ozone; PM_10_, particulate matter < 10 μm in size; PM_2.5,_ particulate matter < 2.5 μm in size; SO_2_, sulfur dioxide; THC, total hydrocarbons.
