# Supplementary material for: Long-term ambient hydrocarbons exposure and incidence of ischemic stroke
Source: PLoS One. 2019 Dec 4;14(12):e0225363. doi: 10.1371/journal.pone.0225363 (PMC6892494; doi:10.1371/journal.pone.0225363)
Supplement: S2 Fig — (DOCX) [file pone.0225363.s004.docx]

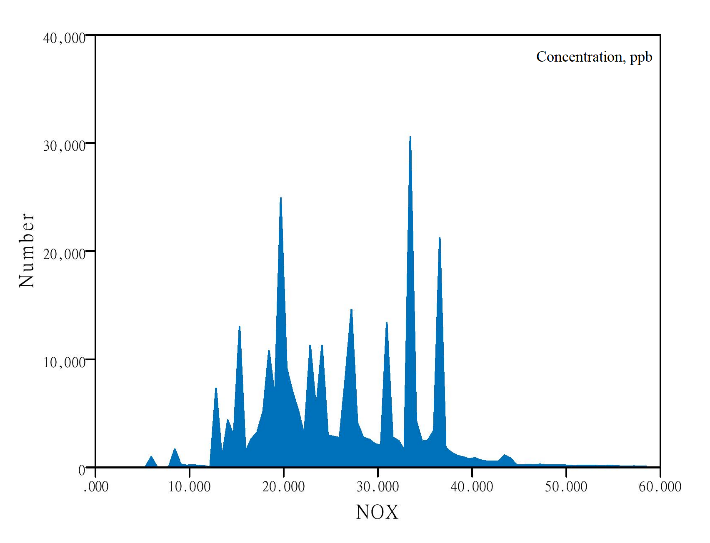

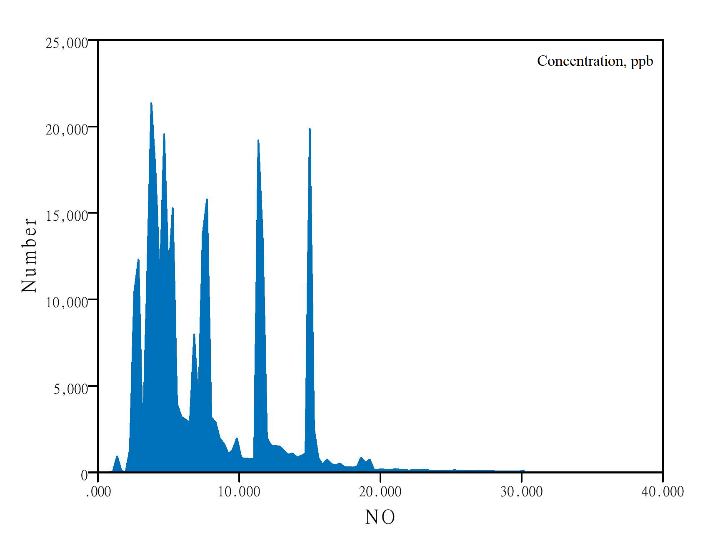


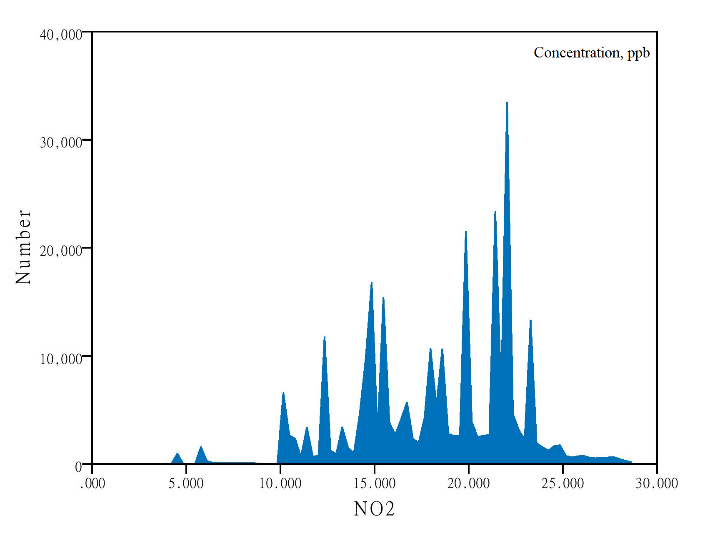

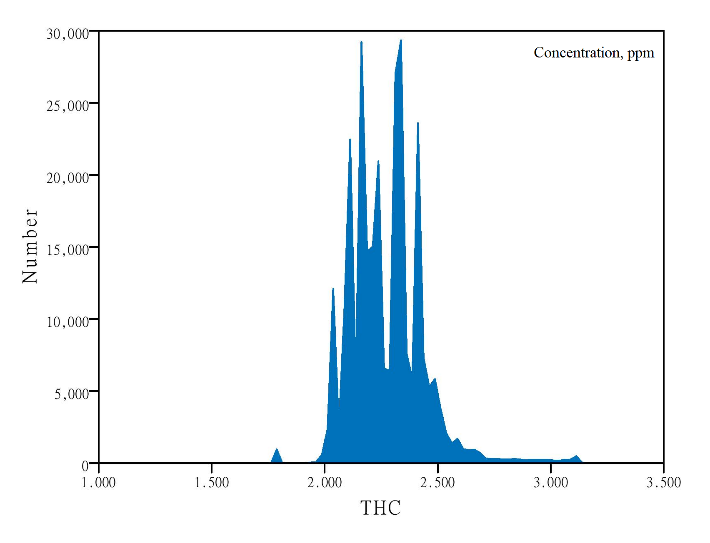


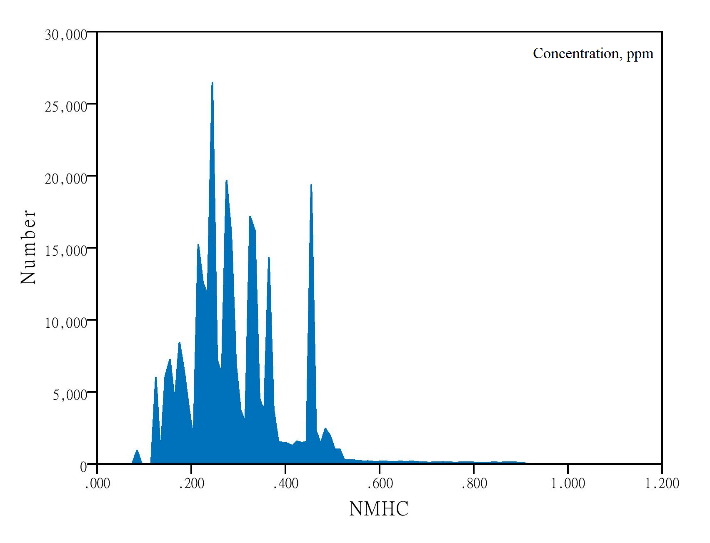

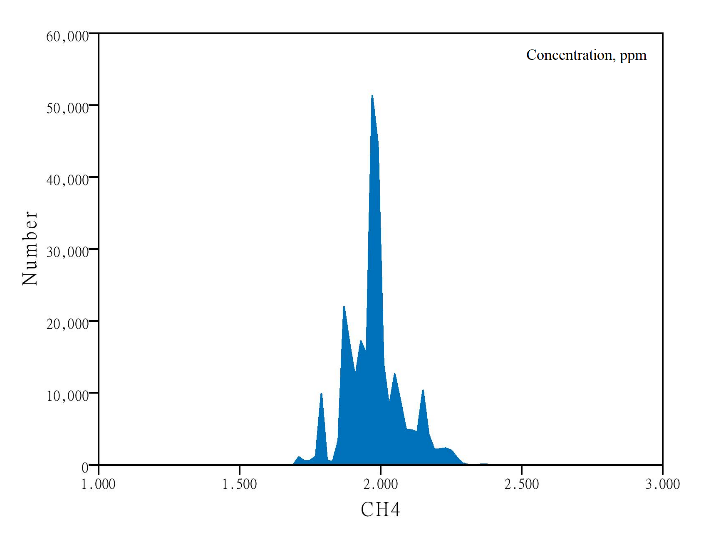


**S2 Fig. Distributions of the daily average concentrations of air pollutants (NO_X_, NO, NO_2_, THC, NMHC, and CH_4_) over 10-year exposure period**
