# Supplementary material for: Long-term ambient hydrocarbons exposure and incidence of ischemic stroke
Source: PLoS One. 2019 Dec 4;14(12):e0225363. doi: 10.1371/journal.pone.0225363 (PMC6892494; doi:10.1371/journal.pone.0225363)
Supplement: S3 Fig — The tertile values, in ppm, are as follows: T1: < 2.18, T2: ≥ 2.18 and < 2.33, and T3: ≥ 2.33. (DOCX) [file pone.0225363.s005.docx]

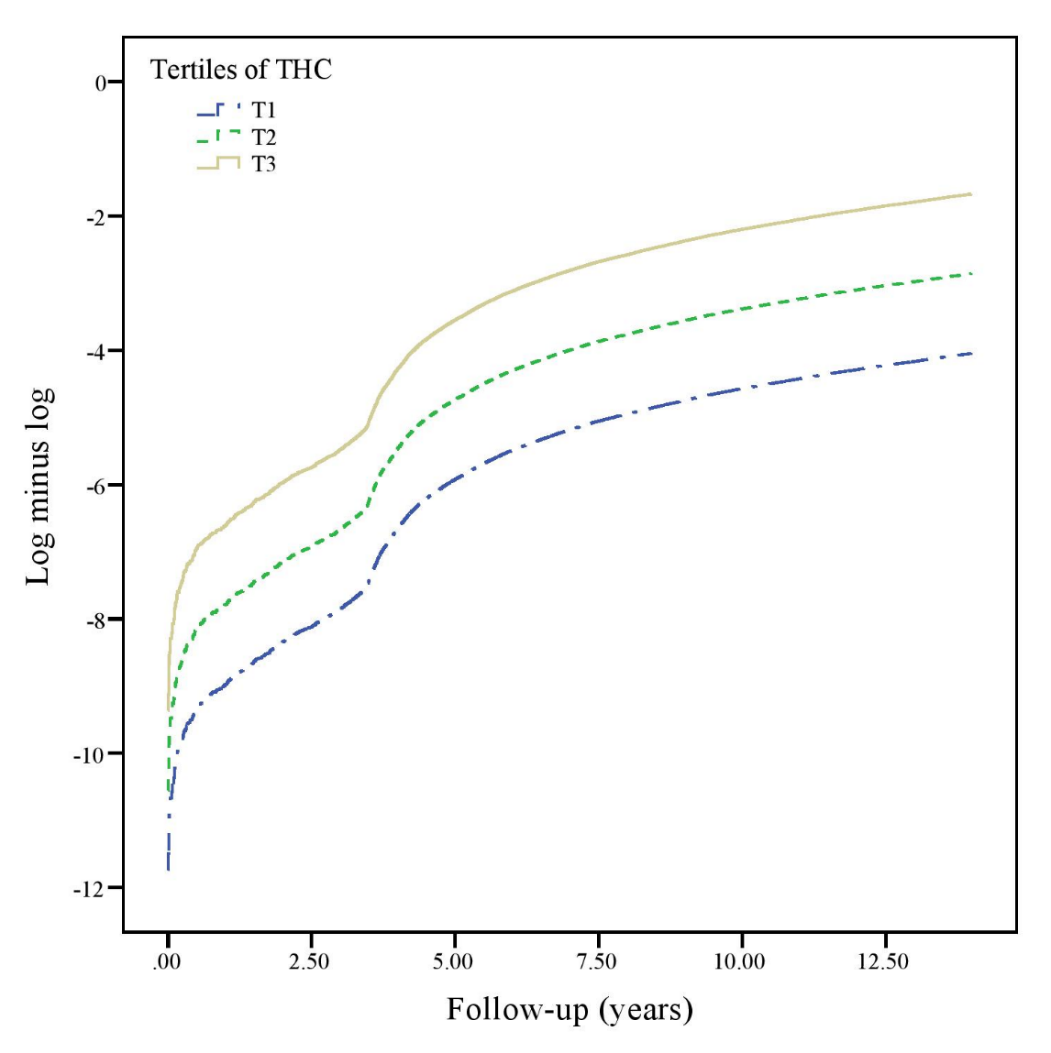


**S3 Fig. The plot of log (−log (survival function)) versus log of survival time in THC**

The tertile values, in ppm, are as follows: T1: < 2.18, T2: ≥ 2.18 and < 2.33, and T3: ≥ 2.33.
