# Supplementary material for: Long-term ambient hydrocarbons exposure and incidence of ischemic stroke
Source: PLoS One. 2019 Dec 4;14(12):e0225363. doi: 10.1371/journal.pone.0225363 (PMC6892494; doi:10.1371/journal.pone.0225363)
Supplement: S4 Fig — The tertile values, in ppm, are as follows: T1: < 0.25, T2: ≥ 0.25 and < 0.33, and T3: ≥ 0.33. (DOCX) [file pone.0225363.s006.docx]

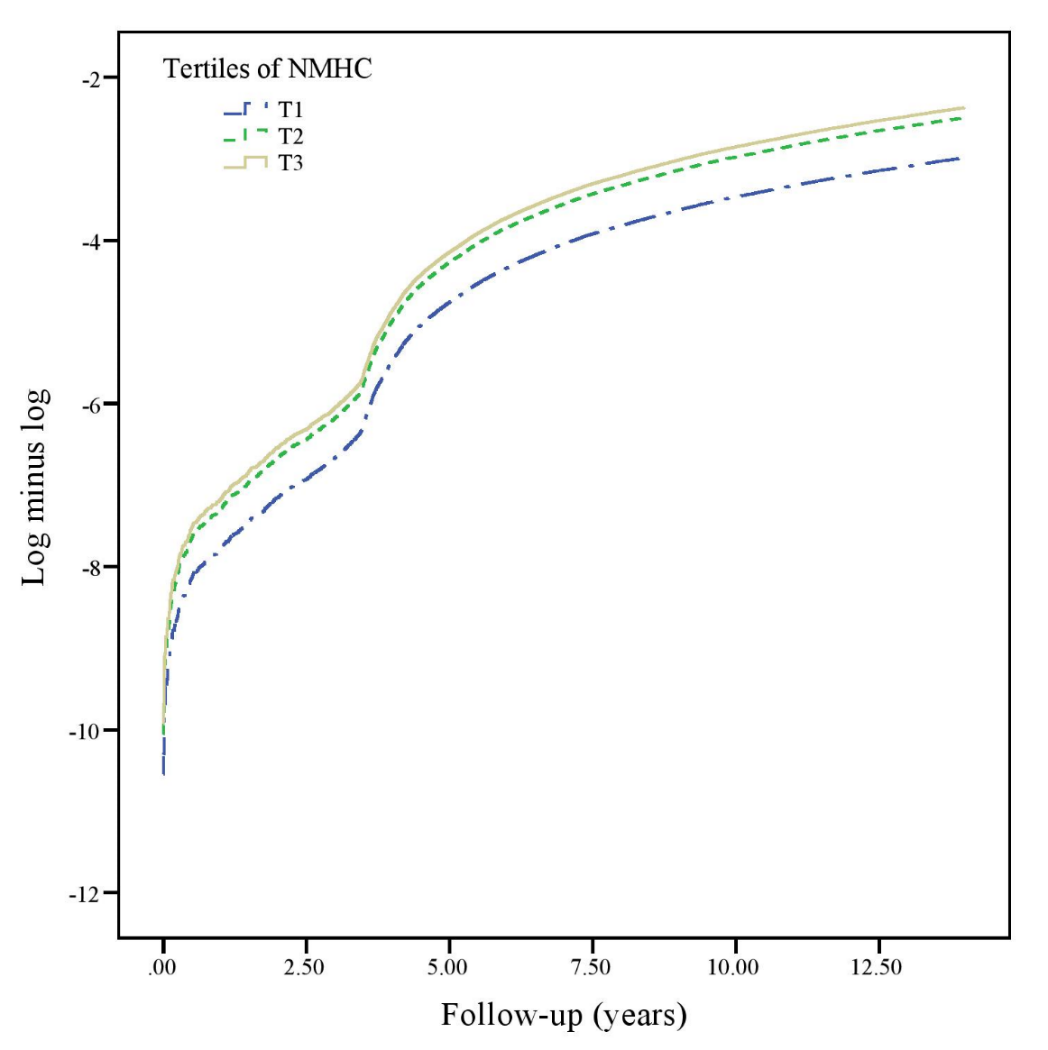


**S4 Fig. The plot of log (−log (survival function)) versus log of survival time in NMHC**

The tertile values, in ppm, are as follows: T1: < 0.25, T2: ≥ 0.25 and < 0.33, and T3: ≥ 0.33.
