# Supplementary material for: Long-term ambient hydrocarbons exposure and incidence of ischemic stroke
Source: PLoS One. 2019 Dec 4;14(12):e0225363. doi: 10.1371/journal.pone.0225363 (PMC6892494; doi:10.1371/journal.pone.0225363)
Supplement: S5 Fig — Cumulative incidence of ischemic stroke for individuals among tertiles of pollutant categories: (A) THC and (B) NMHC. The tertile values, in ppm (THC, NMHC), are as follows: THC (T1: < 2.18, T2: ≥ 2.18 and < 2.33, and T3: ≥ 2.33); NMHC (T1: < 0.25, T2: ≥ 0.25 and < 0.33, and T3: ≥ 0.33). (DOCX) [file pone.0225363.s007.docx]

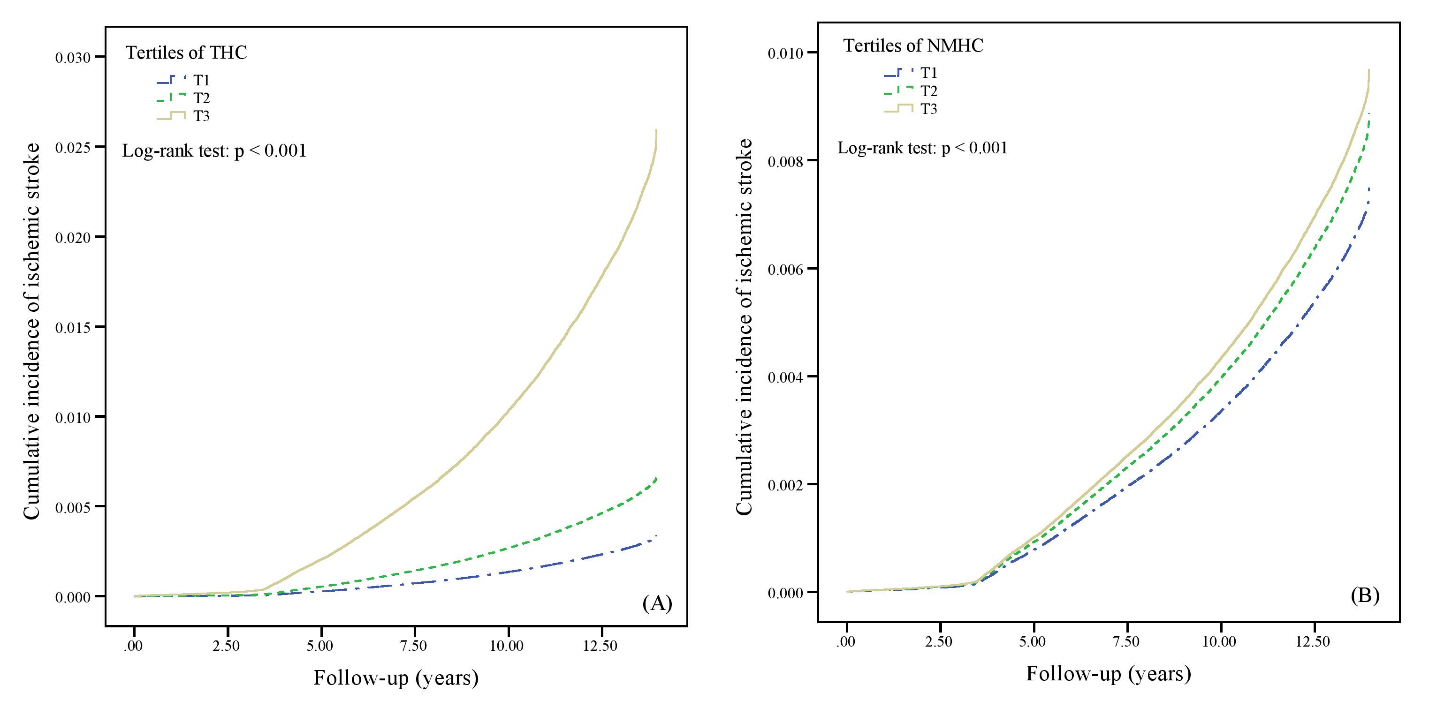


**S5 Fig. Cumulative incidence of ischemic stroke for individuals among tertiles of pollutant categories: (A) THC and (B) NMHC**

The tertile values, in ppm (THC, NMHC), are as follows:

THC (T1: < 2.18, T2: ≥ 2.18 and < 2.33, and T3: ≥ 2.33); NMHC (T1: < 0.25, T2: ≥ 0.25 and < 0.33, and T3: ≥ 0.33).
